# Relative age effect across the talent identification process of youth female soccer players in the United States: Influence of birth year, position, biological maturation, and skill level

**DOI:** 10.5114/biolsport.2024.136085

**Published:** 2024-05-17

**Authors:** Laura Finnegan, Mirelle van Rijbroek, José M. Oliva-Lozano, Rick Cost, Matthew Andrew

**Affiliations:** 1 Football Research Group, Department of Sport and Exercise Science, South East Technological University, Ireland; 2 United States Soccer Federation, Chicago, Illinois, USA; 3 Department of Sport and Exercise Science, Manchester Metropolitan University Institute of Sport, Manchester, UK

**Keywords:** Selection, Development, Youth National Team, Club, Soccer, Talent

## Abstract

The aims of the study were to examine the relative age effect (RAE) in youth female soccer players in the United States (US) and the influence of birth year, playing position, estimated maturation and skill ratings. The sample consisted of 3,364 youth female soccer players who were active in the 2021–2022 US soccer season across three main stages of the talent identification (TID) process for Youth National Team (YNT) players (i.e., *Club, TID Center*, and *YNT*). A prevalent RAE for players born in Q1 was present in the full sample. A significant prevalence for Q1 players were identified for both *Club* and *TID* Center, but not *YNT*. A significant RAE prevalence for Q1 players was identified for most of the age groups from U13–U18 at *Club* (except U18) and *TID Center* (except U17). Significant RAEs prevalence for players born in Q1 were found in Goalkeepers, Center Backs, Midfielders, and Center Forwards at *Club* and *TID* Center (except Wide Forwards). The data identified a consistent RAE prevalence for Q1 players in early and on-time-maturers across all levels. An even birthdate spread was evident in YNT with a prevalence for Q4 players and a higher percentage of late-maturers than elsewhere in the TID process. Results reinforce evidence indicating RAEs still exist in soccer, yet show for the first time within a youth female soccer TID process, the influence of contextual factors on the prevalence of RAE. This information can be used to advance TID and development across the US soccer landscape.

## INTRODUCTION

Soccer is one of the most popular female sports worldwide [1]. In the United States (US) this popularity is rising exponentially due to success at domestic and international levels [1]. To continue this success, soccer-teams and -nations identify talented youth players that show the potential to enter a high-performance programme [2]. While research that aims to provide evidenced-based information to support female soccer is slowly rising [3], there is an underrepresentation of female-only research in talent identification (TID) [4], thus calls for female-specific research have been made [2].

One factor that is known to influence TID is when an athlete is born within a selection year, also known as the Relative Age Effect (RAE) [5]. RAE refers to the (dis)advantage of chronological age differences between individuals within annually age-grouped cohorts, with those born close to the start of a cut-off (first quartile of year) date almost 12 months older compared to those born later in the cut-off (fourth quartile of year) date. Due to the subjective nature of TID, scouts may (un)consciously judge older players as more talented than their younger peers and thus they may be more likely to select them into high-performance-environments [6]. This judgement may be associated with the older athletes possessing performance advantages (e.g., anthropometrical) that obscures a scout’s ability to observe other predictors such as technical/perceptual skills [7].

Research on RAE in soccer has primarily focused on male soccer players, with most studies indicating this effect still persists at youth and professional levels [8, 9]. The small sample of studies examining RAE in female soccer players have produced inconsistent findings [10]. For example, no RAEs were observed in youth or senior soccer players that competed in European Championships qualification campaigns [11], or senior players representing their nation at Olympic Games [12]. However, retrospective analyses have indicated RAEs in national female soccer players of youth World Cups, particularly midfielders, but did not translate to senior levels [13]. These comparisons between-soccer-nations indicate the impact of global contextual factors on the level of RAE in female soccer, such as competition level, birth year, and playing position, and should continue to be studied [13].

Individual-soccer-nation examinations of RAE in female soccer are also historically mixed, with no RAEs observed in league players in France [14] and Brazil [15], or national players in Switzerland [16], but RAEs have been reported in youth players in China [17], and league players in Spain [18], Italy [19], and Japan [20]. Götze and Hoppe [21] reported RAEs for league players in Germany but not youth national players. Whereas Brustio et al. [22] reported RAEs for youth national players, this did not translate to the senior level. It has been suggested that the soccer environments of the individual nations may underpin inconsistencies in RAEs [23, 24]. Soccer nations differ based on demographical (population, size, depth of competition, resources, participation [25]), sociocultural (facilities, schooling, registered coach numbers, hours in practice, socioeconomic status [2, 26, 27]) and TID (scout numbers, players recruited/released, staff roles, objectives [27]) factors. So, the soccer environment should be considered when examining RAE in female soccer players.

**FIG. 1:**
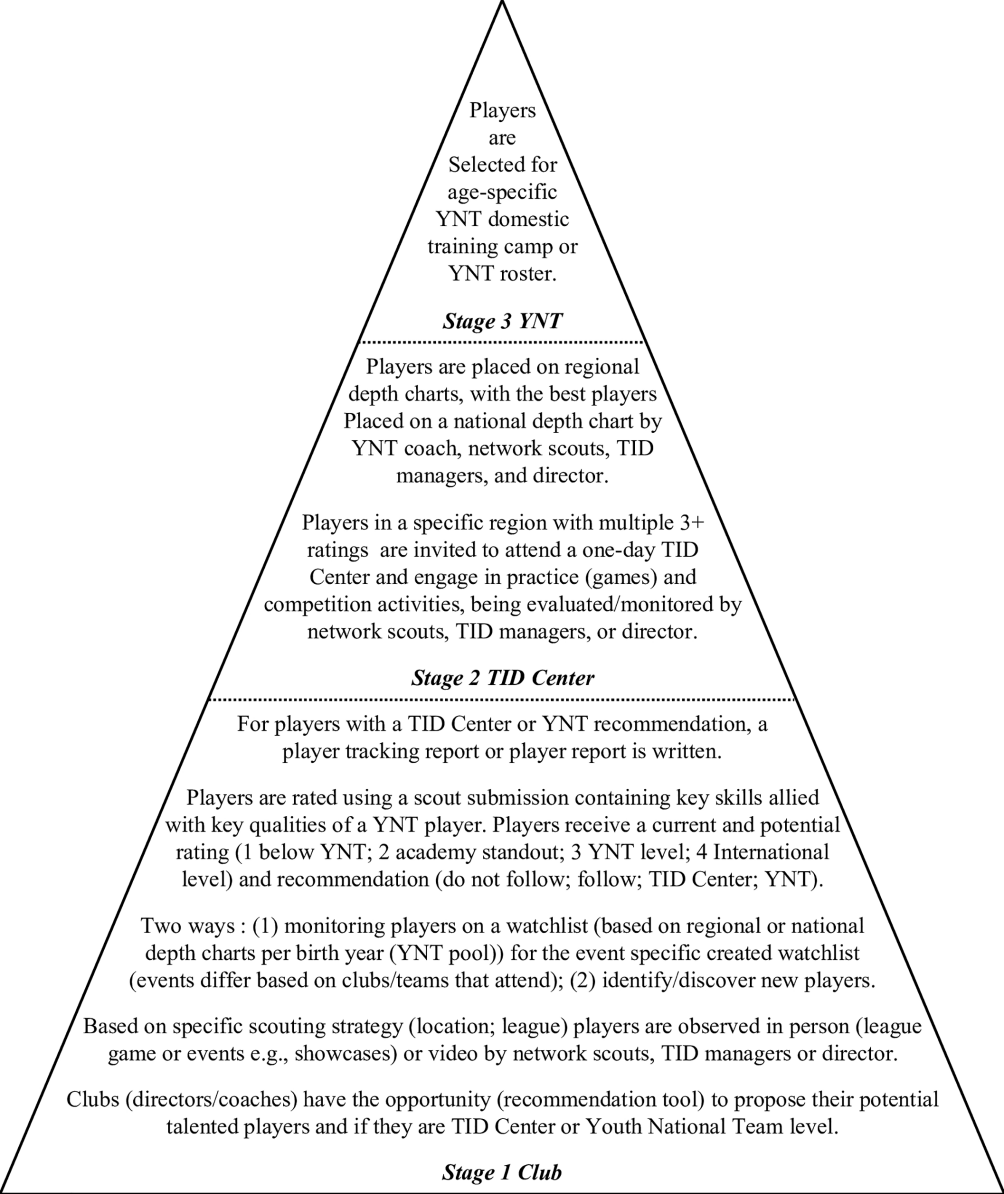
Three key stages of the talent identification process for Youth National Team players outlined by US Soccer.

The US are one of the most successful female soccer-nations at senior (4 × FIFA Word Cups; 4 × Olympic Gold Medals; 9 × CONCACAFW-Championships) and youth (3 × FIFA Word Cups; 15 × CONCACAFW-Championships) levels. To continue their success, the US Soccer Federation (USSF) utilises its TID processes which is multi-layered (an outline of the stages can be seen in Figure 1). There are three main stages: (1) *Club*, based on a specific scouting strategy (location; league), players are observed in their club environment (league/events) by Youth National Team (YNT) network scouts, TID manager(s)/director, and clubs recommending players (via a recommendation tool). Playing position, estimated biological maturation, birthdate/quartile, rating of current performance and potential ability, and recommendations compared to US YNT key qualities are recorded; (2) *TID Center*, based on these reports, high-rated players in each region attend a single day of training and competition (vs. each other and/or boys’ teams), again being evaluated/monitored. Players are placed on regional depth charts, with the players with the highest potential ability on a national depth chart; (3) *YNT*, players are selected for agespecific domestic training camps or rosters. Previous examinations of US youth female soccer have reported RAEs in both youth club-level soccer players between 2012–2013 [28] and U17 national team players [24]. However, it is currently unknown whether RAEs still exist following an exponential rise in participation rates and/or whether it translates to younger and older national players. This historical data may not provide a full picture of the current US female soccer environment and the effects mediated by birth year, maturation, and playing status. Therefore, it is necessary to explore RAEs across the TID process and identify if/where the RAE extent occurs between levels [11].

The present study aimed to examine the influence of RAE in US youth female soccer players across the TID process, and to identify if these are moderated by birth year, playing position, estimated maturation, and skill. Given the limited and mixed literature examining RAEs in female soccer players, we did not make any a priori hypotheses.

## MATERIALS AND METHODS

### Participants

Birthdates of 3,364 youth female soccer players across the 2021–2022 season were analysed across three stages of the TID process. For *Club* (Stage 1, Figure 1), birthdates were collected from 1,940 players and were categorised by birth year (BY), playing position (as per the player profiles outlined by US Soccer), estimated maturation, and skill rating. For *TID Center* (Stage 2, Figure 1), birthdates were collected from 1,191 players that attended a YNT TID Center event. For *YNT* (Stage 3, Figure 1), birthdates were collected from 233 players who were selected to be part of a domestic training camp and/or roster. Skill ratings were not collected for *YNT* as players are considered the most skilled within their age group. The study was conducted in accordance with the declaration of Helsinki, and ethical approval was gained from an institutional ethics committee (2023-50926-40701).

### Procedure

For *Club,* data was taken from 4,818 (2.4 ± 2.0 per player) tracking/ player reports completed by 66 US YNT network scouts, TID manager(s) and director following observations of the players within their club setting (e.g., league game; Figure 1). Scout observations were either independent or with another scout, TID manager, or director, where estimated maturation and skill rating were agreed upon. The scouts had between 1–31 years of experience, 1–21 years of which were specifically for US YNT players. Many scouts held USSF coaching licenses (e.g., USSF ‘A’ and ‘B’), and had completed educational courses (including on the topics of TID and the maturation and development of female soccer players) delivered by the research team. Biological maturation was recorded through subjective estimations by the scouts, and consistent with Romann et al. [29] were classified into early-, on-time- and late-maturing players. An interclass correlation coefficient (ICC) was used for players with 2 or more observations that indicated an ICC of 0.61 for inter-interindividual and 0.85 for intra-individual, demonstrating moderate-good reliability. Furthermore, skill was recorded through subjective assessments by the scouts, whereby they compared the players’ soccerspecific skills to the US YNT key qualities and provided a skill rating. They were classified into below YNT, follow (TID recommendation), and YNT level. Skill was subdivided into current (present) and potential (possible) performance. Playing position was categorised based on most appearances. For both estimated biological maturation and skill ratings, we created mean values from all observations within the season. For *TID Center,* data was taken from the player reports completed by 61 US YNT network scouts, TID manager(s) and director following the event. Observations were conducted as a small group including scouts, TID manager(s) and director.

The birth month for each player was used to define birth quartile (BQ) and half-year distribution per semester (BS) [5]. In line with the changes in cut-off dates proposed in 2017 by US Soccer [30], we adopted cut-off dates of: Q1 = Jan-Mar; Q2 = Apr-Jun; Q3 = Jul-Sep; Q4 = Oct-Dec, and semesters: S1 = Jan-Jun; S2 = Jul-Dec. For players born 1999–2004, we adopted pre-2017 cut-off dates of: Q1 = Aug-Oct; Q2 = Nov-Jan; Q3 = Feb-Apr; Q4 = May-Jul, and semesters: S1 = Aug-Jan; S2 = Feb-Jul. A failure to be aware of these changes could lead to skewed results within large-scale RAE studies [31].

### Data analysis

The Chi-squared (*χ*^2^) test was used to assess differences between observed and expected birthdate distributions across BQs for: each birth year (BY) irrespective of time point; and each BY, playing position, current performance and potential ability ratings per time point. Expected BQs were taken from the National Center for Health Statistics in the Center for Disease Control and Prevention (www.cdc.com) and reflected the average population BQs for the US from 1999–2009 (oldest-youngest within sample). BQs were identified as: Q1 = 24.1%; Q2 = 24.7%; Q3 = 26.3%; Q4 = 24.8%. Odds ratios (ORs) and 95% confidence intervals (95% CI) were calculated to compare the odds of the frequency of a BQ/S to another with a reference group, consisting of the youngest players (Q4 or S2 respectively). An OR of 1.0 indicated that the frequency is equal in both BQs/BSs whilst an OR of 2.0 indicated that the frequency of one BQ/BS is twice as high as the other [10; 21]. ORs were considered significant if the 95% CI range did not include a value < 1.00. Furthermore, effect sizes (ES) were calculated through Cohen’s w [32] and interpreted as small effect (w < 0.30), medium effect (w = 0.30–0.50), and large effect (w > 0.50). Alpha was set at p < 0.05. Data were analysed via SPSS Statistics (IBM, Chicago, US).

## RESULTS

### Overall

The distribution of BQs across *Club, TID Center*, and *YNT* are presented in Table 1. Results show RAE prevalence in the full sample (Q1 = 34.8%, Q2 = 28.6%, Q3 = 22.8%, Q4 = 13.8%; *χ*^2^ (3, *n* = 3,364) = 10.8, *p* = 0.01, *w* = 0.33). Overall, for *Club* and *TID Center*, there was a significant RAE, with Q1 players being overrepresented. This RAE effect was lesser at *YNT*. To gain further insights at *YNT*, that dataset was compared to both *Club* and *TID Center*, showing significant differences from both, *p* = 0.02 and *p* = 0.01, respectively (Table 1).

**TABLE 1:** Birth quartile distribution by birth year.

	n	Birthdate Distribution (%)				Odds Ratio (95% CI)				χ^2^	p	w

		Q1	Q2	Q3	Q4	Q1 vs. Q2	Q1 vs. Q3	Q1 vs. Q4	S1 vs. S2			
*Club*												

2004 (U18)	247	83 (33.6)	59 (23.9)	47 (19.0)	58 (23.5)	1.4 (0.7–3.1)	1.9 (0.9–4.2)	1.5 (0.7–3.2)	1.4 (0.8, 2.5)	5.87	0.12	0.24 (Small)

2005 (U17)	383	134 (35.0)	104 (27.2)	98 (25.6)	47 (12.3)	1.3 (0.6–2.8)	1.5 (0.7–3.2)	2.9 (1.2–6.9)	1.7 ()	11.50^*^	0.01	0.34 (Medium)

2006 (U16)	448	163 (36.4)	127 (28.3)	111 (24.8)	47 (10.5)	1.3 (0.6–2.8)	1.6 (0.8–3.4)	3.6 (1.5–8.7)	1.9 (1.1–3.4)	15.13^*^	0.00	0.39 (Medium)

2007 (U15)	379	127 (33.5)	126 (33.2)	77 (20.3)	49 (12.9)	1.0 (0.5–2.2)	1.8 (0.8–3.9)	2.7 (1.1–6.3)	2.1 (1.2–3.7)	13.67^*^	0.00	0.37 (Medium)

2008 (U14)	341	128 (37.5)	89 (26.1)	73 (21.4)	51 (15.0)	1.5 (0.7–3.1)	1.6 (0.7–3.3)	2.6 (1.1–5.8)	1.8 (1.0–3.2)	12.32^*^	0.01	0.35 (Medium)

2009 (U13)	142	56 (39.4)	46 (32.4)	35 (24.6)	5 (3.5)	1.2 (0.6–2.6)	1.7 (0.8–3.7)	11.6 (3.4–39.6)	2.7 (1.5–4.8)	30.52^*^	0.00	0.55 (Large)

All	1940	691 (35.6)	551 (28.4)	441 (22.7)	257 (13.2)	1.3 (2.7–0.6)	1.7 (0.8–3.7)	2.8 (1.2–6.5)	1.9 (1.1–3.3)	11.96^*^	0.01	0.35 (Medium)

*TID Center*												

2004 (U18)	13	2 (15.4)	2 (15.4)	5 (38.5)	4 (30.8)	1.0 (0.4, 2.5)	0.4 (0.2–1.0)	0.5 (0.2–1.2)	0.5 (0.2–0.8)	13.75^*^	0.00	0.37 (Medium)

2005 (U17)	42	10 (23.8)	14 (33.3)	8 (19.0)	10 (23.8)	0.7 (0.3, 1.6)	1.4 (0.6–3.1)	1 (0.5–2.3)	1.4 (0.7–2.4)	5.06	0.17	0.23 (Small)

2006 (U16)	292	101 (34.6)	83 (28.4)	76 (26.0)	32 (11.0)	1.2 (0.6, 2.6)	1.5 (0.7–3.1)	3.2 (1.3–7.8)	1.8 (1–3.1)	12.81^*^	0.01	0.36 (Medium)

2007 (U15)	387	130 (33.6)	129 (33.3)	83 (21.4)	45 (11.6)	1.0 (0.5, 2.2)	1.7 (0.8–3.7)	3.0 (1.2–7.1)	2.1 (1.2–3.8)	14.68^*^	0.00	0.38 (Medium)

2008 (U14)	434	167 (38.5)	116 (26.7)	91 (21.0)	60 (13.8)	1.5 (0.7, 31)	2.0 (0.9–4.3)	2.9 (1.2–6.6)	2 (1.1–3.5)	14.71^*^	0.00	0.30 (Medium)

2009 (U13)	23	10 (43.5)	6 (26.1)	6 (26.1)	1 (4.3)	1.7 (0.8, 3.6)	1.8 (0.9–3.8)	10.4 (3.3–32.5)	2.4 (1.3–4.3)	32.64^*^	0.00	0.57 (Large)

All	1191	421 (35.3)	350 (29.4)	269 (22.6)	152 (12.8)	1.2 (0.6, 2.6)	1.7 (0.8–3.7)	2.8 (1.2–6.6)	1.9 (1.1–3.4)	12.43^*^	0.01	0.35 (Medium)

*YNT*												

^a^1999–2001 (U23)	44	10 (22.7)	13 (29.5)	12 (27.3)	9 (20.5)	0.7 (0.3–1.6)	1.3 (0.6–3.1)	1.4 (0.6–3.2)	1.1 (0.7–2.0)	1.80	0.62	0.13 (Small)

^a^2002–2004 (U20)	60	17 (28.3)	12 (20.0)	12 (20.0)	19 (31.7)	1.2 (0.5–3.0)	0.5 (0.3–1.2)	1.3 (0.6–3.0)	1.0 (0.6–1.7)	5.06	0.17	0.22 (Small)

2005 (U17)	47	13 (27.7)	13 (27.7)	10 (21.3)	11 (23.4)	1.0 (0.5–2.2)	1.4 (0.6–3.1)	1.2 (0.6–2.7)	1.3 (0.7–2.3)	1.93	0.59	0.14 (Small)

2006 (U16)	23	5 (21.7)	4 (17.4)	10 (43.5)	4 (17.4)	1.5 (0.7–3.2)	1.5 (0.7–3.4)	0.9 (0.4–2.0)	0.7 (0.4–1.2)	15.85^*^	0.00	0.39 (Medium)

2007 (U15)	59	15 (25.4)	21 (35.6)	12 (20.3)	11 (18.6)	0.8 (0.4–1.7)	0.9 (0.4–2.0)	1.1 (0.5–2.6)	1.6 (0.9–2.9)	7.80^*^	0.05	0.28 (Small)

All	233	60 (25.8)	63 (27.0)	56 (24.0)	54 (23.2)	0.7 (0.3–1.6)	1.3 (0.6–3.1)	1.4 (0.6–3.2)	1.2 (0.7–2.0)	0.64	0.89	0.08 (Small)

TID = talent identification; YNT = Youth National Team; Q1 = Jan-Mar; Q2 = Apr-Jun; Q3 = Jul-Sep; Q4 = Oct-Dec, S1 = Jan-Jun, S2 = Jul-Dec, aQ1 = Aug-Oct; Q2 = Nov-Jan; Q3 = Feb-Apr; Q4 = May-Jul, and semesters: S1 = Aug-Jan; S2 = Feb-Jul, χ^2^ = Chi-squared,

* Significant at an alpha level of p < 0.05, w = Cohen’s w effect size.

### Birth year

The frequency and percentage distributions of players’ BQs for BY are provided in Table 1. In *Club*, the chi-squared indicated significant deviations for U13–U17, with Q1 players being over-represented. Analysis further revealed that although Q1 were over-represented, there was no significant RAE for U18. Within *TID* Centers, the chisquared indicated significant deviations for U13–U16, with Q1 players being over-represented, and the ORs remaining relatively similar across all BYs. However, for U18, Q3 players were over-represented, with the representation of Q4 players being larger than Q1. Analysis further indicated that for U17, though Q2 were over-represented, a significant RAE did not exist. For *YNT*, the chi-squared indicated significant deviations for U15–U16 only, with Q3 players being overrepresented for U16 and Q2 players for U15. For all other BYs, BQs were relatively evenly distributed.

### Position

The frequency and percentage distributions of players’ BQs for position are presented in Table 2. In *Club*, for full backs, the largest distribution was observed in Q1, yet this did not reach significance. Q1 players represented the largest distribution for all positions, with a progressive decline from Q1–Q4. OR analysis indicated that RAE was highest for the center backs. When analysed by BY, within 2009, from the seven goalkeepers, one was born in S2. In *TID Center*, for full backs and wide forwards, the largest distribution was observed in Q1, yet this did not reach significance. Q1 players also represented the largest distribution for most other positions. For these positions, OR analysis indicated that RAE was highest for center backs and center forwards, remaining relatively similar in goalkeepers and midfielders. For goalkeepers, the largest distribution was Q2. For *YNT*, there was a significant RAE for goalkeepers only, with Q2 and Q3 players being over-represented and Q4 being under-represented. A mixed pattern emerged from the other positional data, with Q4 being most represented in midfielders, Q3 with center backs, and Q2 with wide forwards and center forwards.

**TABLE 2:** Birth quartile distribution by position.

	n	Birthdate Distribution (%)				Odds Ratio (95% CI)				χ^2^	p	w

		Q1	Q2	Q3	Q4	Q1 vs. Q2	Q1 vs. Q3	Q1 vs. Q4	S1 vs. S2			
*Club*												

Goalkeeper	177	63 (35.6)	62 (35.0)	33 (18.6)	19 (10.7)	1.0 (0.5–2.2)	2.1 (0.9–4.6)	3.4 (1.4–8.3)	2.5 (1.4–4.5)	20.06^*^	0.00	0.45 (Medium)

Full Backs	221	72 (32.6)	56 (25.3)	55 (24.9)	38 (17.2)	1.3 (0.6–2.8)	1.4 (0.7–3.1)	2.0 (0.9–4.4)	2.1 (1.1–3.7)	5.42	0.14	0.23 (Small)

Center Backs	286	125 (43.7)	86 (30.1)	51 (17.8)	24 (8.4)	1.5 (0.7–3.1)	2.7 (1.2–5.8)	5.4 (2.1–13.5)	3 (1.6–5.3)	30.71^*^	0.00	0.51 (Large)

Midfields	701	248 (35.4)	187 (26.7)	167 (23.8)	99 (14.1)	1.4 (0.6–2.9)	1.6 (0.8–3.5)	2.6 (1.1–5.9)	1.7 (1.0–3.0)	10.32^*^	0.02	0.32 (Medium)

Wide Forwards	330	106 (32.1)	97 (29.4)	79 (23.9)	48 (14.5)	1.1 (0.5–2.4)	1.5 (0.7–3.2)	2.3 (1.0–5.3)	1.7 (1.0–2.9)	8.05^*^	0.05	0.28 (Small)

Center Forward	221	74 (33.5)	62 (28.1)	56 (25.3)	29 (13.1)	1.2 (0.6–2.6)	1.4 (0.7–3.1)	2.6 (1.1–6.2)	1.7 (1.0–2.9)	9.69^*^	0.02	0.31 (Medium)

*TID Center*												

Goalkeeper	114	34 (29.8)	41 (36.0)	27 (23.7)	12 (10.5)	0.8 (0.4–1.8)	1.4 (0.6–3.0)	2.9 (1.2–7.2)	2.0 (1.1–3.6)	15.02^*^	0.00	0.39 (Medium)

Full Backs	162	49 (30.2)	47 (29.0)	35 (21.6)	31 (19.1)	1.1 (0.5–2.3)	1.5 (0.7–3.3)	1.6 (0.7–3.6)	1.5 (0.9–2.7)	4.45	0.22	0.21 (Small)

Center Backs	182	78 (42.9)	51 (28.0)	35 (19.2)	18 (9.9)	1.6 (0.8–3.3)	2.4 (1.1–5.3)	4.5 (1.8–10.9)	2.6 (1.4–4.6)	25.97^*^	0.00	0.51 (Large)

Midfields	367	128 (34.9)	106 (28.9)	88 (24)	45 (12.3)	1.2 (0.6–2.6)	1.6 (0.7–3.4)	2.9 (1.2–6.9)	1.8 (1.0–3.2)	12.06^*^	0.01	0.35 (Medium)

Wide Forwards	190	58 (30.5)	50 (26.3)	49 (25.8)	33 (17.4)	1.2 (0.6–2.6)	1.3 (0.6–2.8)	1.8 (0.8–4.1)	1.4 (0.8–2.4)	4.02	0.26	0.20 (Small)

Center Forward	134	53 (39.6)	46 (34.3)	28 (20.9)	7 (5.2)	1.2 (0.6–2.4)	2.1 (1.0–4.4)	7.8 (2.7–22.9)	3.0 (1.6–5.4)	30.29^*^	0.00	0.55 (Large)

*YNT*												

Goalkeeper	24	7 (29.2)	8 (33.3)	8 (33.3)	1 (4.2)	0.9 (0.4–1.9)	1.0 (0.5–2.0)	7.2 (2.2–23.0)	1.7 (1.0–3.1)	23.03^*^	0.00	0.48 (Medium)

Full Backs	30	8 (26.7)	9 (30.0)	5 (16.7)	8 (26.7)	0.9 (0.4–2.0)	1.7 (0.8–4.0)	1.0 (0.5–2.2)	1.4 (0.8–2.4)	5.08	0.67	0.22 (Small)

Center Backs	39	10 (25.6)	10 (25.6)	11 (28.2)	8 (20.5)	1.0 (0.5–2.2)	1.0 (0.5–2.1)	1.3 (0.6–2.9)	1.1 (0.6–1.9)	1.01	0.80	0.10 (Small)

Midfields	79	19 (24.1)	17 (21.5)	19 (24.1)	24 (30.4)	1.1 (0.5–2.6)	1.1 (0.5–2.4)	0.8 (0.4–1.8)	0.9 (0.5–1.5)	1.86	0.60	0.14 (Small)

Wide Forwards	38	10 (26.3)	12 (31.6)	7 (18.4)	9 (23.7)	0.9 (0.4–1.8)	1.6 (0.7–3.5)	1.1 (0.5–2.5)	1.4 (0.8–2.5)	4.56	0.21	0.21 (Small)

Center Forward	23	6 (26.1)	7 (30.4)	6 (26.1)	4 (17.4)	0.9 (0.4–1.9)	1.1 (0.0–2.4)	1.6 (0.7–3.6)	1.4 (0.8–2.4)	3.88	0.28	0.31 (Medium)

TID = talent identification; YNT = Youth National Team; Q1 = Jan-Mar; Q2 = Apr-Jun; Q3 = Jul-Sep; Q4 = Oct-Dec, S1 = Jan-Jun, S2 = Jul-Dec, aQ1 = Aug-Oct; Q2 = Nov-Jan; Q3 = Feb-Apr; Q4 = May-Jul, and semesters: S1 = Aug-Jan; S2 = Feb-Jul, χ^2^ = Chisquared,

* Significant at an alpha level of p < 0.05, w = Cohen’s w effect size.

### Estimated biological maturation

The overall group consisted of 28.9% early-, 60.9% on time-, and 10.2% late-maturers. In *Club*, for late-maturers, the largest distribution was observed in Q3, yet this did not reach significance. Q1 players represented the largest BQ for both the on-time- and earlymaturers, which was in line with the general BQ statistics. The fewest players deemed to be early- and on-time-maturers were Q4. OR analysis indicated that RAE was slightly higher for the on-time-, compared to the early-maturers (Table 3). There was a significant difference between BY and estimated maturity ratings (*χ*^2^ (10, *n* = 1930) = 66.87, *p* < .01, *w* = 0.42). For BY2009, 19.9% were deemed late-maturing compared to 2.9% of BY2004. In *TID Center*, 34.9% of Q1 players were early-maturers compared to 7.6% of Q4 players. OR analysis indicated that RAE was higher for the early-maturers compared to the on-time. For *YNT*, overall, there was a statistically significant RAE for all players. But, for earlymaturers Q2 players were over-represented and Q1 on-time players were over-represented. For late-maturers, this was reversed, with Q4 players being over-represented. *YNT* had the highest proportion of late-maturers (14.6%), compared to *Club* (9.5%) and *TID Center* (10.8%).

**TABLE 3:** Birth quartile distribution by estimated maturation.

	n	Birthdate Distribution (%)				Odds Ratio (95% CI)				χ^2^	p	w

		Q1	Q2	Q3	Q4	Q1 vs. Q2	Q1 vs. Q3	Q1 vs. Q4	S1 vs. S2			
*Club*												
Early-Maturers	556	211 (38.0)	158 (28.4)	117 (21.0)	70 (12.6)	1.4 (0.7–2.9)	2.0 (0.9–4.2)	3.1 (1.3–7.2)	2.1 (1.2–3.7)	15.53^*^	0.00	0.39 (Medium)
On-Time-Maturers	1191	430 (36.1)	355 (29.8)	268 (22.5)	138 (11.6)	1.2 (0.6–1.8)	1.8 (0.8–3.8)	3.2 (1.3–7.6)	2 (1.1–3.6)	14.61^*^	0.00	0.38 (Medium)
Late-Maturers	183	45 (24.6)	37 (20.2)	55 (30.1)	46 (25.1)	1.2 (0.6–2.8)	0.9 (0.4–1.9)	1.0 (0.5–2.2)	0.9 (0.5–1.5)	1.37	0.71	0.12 (Small)

*TID Center*												
Early-Maturers	343	129 (37.6)	106 (30.9)	82 (23.9)	26 (7.6)	1.2 (0.6–2.6)	1.7 (0.8–3.6)	5.1 (1.9–13.3)	2.3 (1.3–4.1)	21.26^*^	0.00	0.46 (Medium)
On-Time-Maturers	700	244 (34.9)	210 (30.0)	150 (21.4)	96 (13.7)	1.2 (0.6–2.5)	1.8 (0.8–3.8)	2.6 (1.1–6.1)	1.9 (1.1–3.4)	11.86^*^	0.01	0.34 (Medium)
Late-Maturers	126	43 (34.1)	24 (19.0)	34 (27.0)	25 (19.8)	1.8 (0.8–4.1)	1.4 (0.7–2.9)	1.8 (0.8–3.9)	1.2 (0.7–2.1)	6.49	0.09	0.26 (Small)

*YNT*												
Early-Maturers	33	7 (21.2)	15 (45.5)	6 (18.2)	5 (15.2)	0.5 (0.2–1.0)	1.3 (0.6–2.9)	1.4 (0.6–3.4)	2.1 (1.2–3.7)	24.08^*^	0.00	0.49 (Medium)
On-Time-Maturers	72	23 (31.9)	21 (29.2)	20 (27.8)	8 (11.1)	1.1 (0.5–2.4)	1.3 (0.6–2.7)	3.0 (1.2–7.2)	1.6 (0.9–2.9)	10.99^*^	0.01	0.33 (Medium)
Late-Maturers	18	3 (16.7)	1 (5.6)	4 (22.2)	10 (55.6)	3.1 (1.0–9.3)	0.8 (0.4–1.9)	0.3 (0.1–0.7)	0.3 (0.2–0.6)	55.87^*^	0.00	0.75 (Large)

TID = talent identification; YNT = Youth National Team; Q1 = Jan-Mar; Q2 = Apr-Jun; Q3 = Jul-Sep; Q4 = Oct-Dec, S1 = Jan-Jun, S2 = Jul-Dec, aQ1 = Aug-Oct; Q2 = Nov-Jan; Q3 = Feb-Apr; Q4 = May-Jul, and semesters: S1 = Aug-Jan; S2 = Feb-Jul, χ^2^ = Chisquared,

* Significant at an alpha level of p < 0.05, w = Cohen’s w effect size.

### Skill ratings

**FIG. 2:**
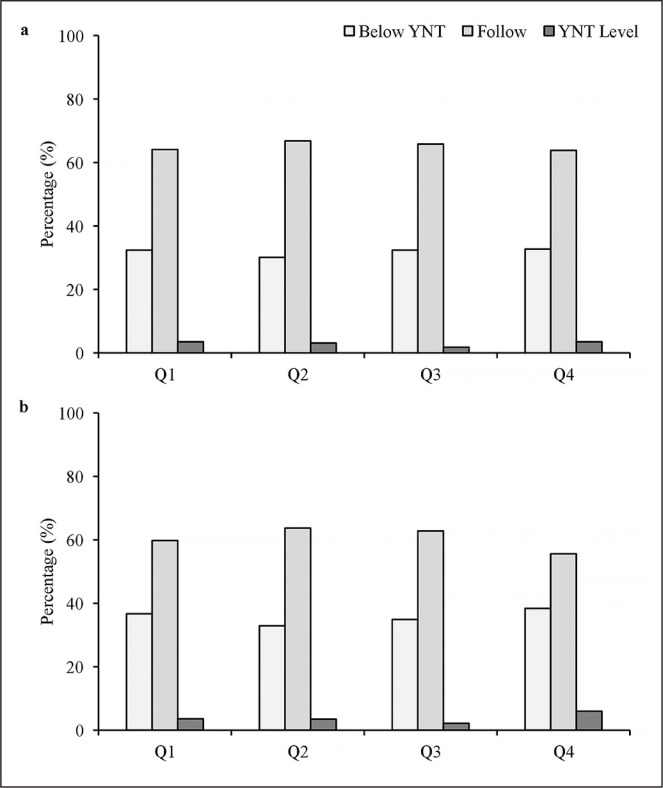
BQ distribution for Club (a) and TID Center (b) presented as a function of overall performance rating. YNT = Youth National Team.

**FIG. 3:**
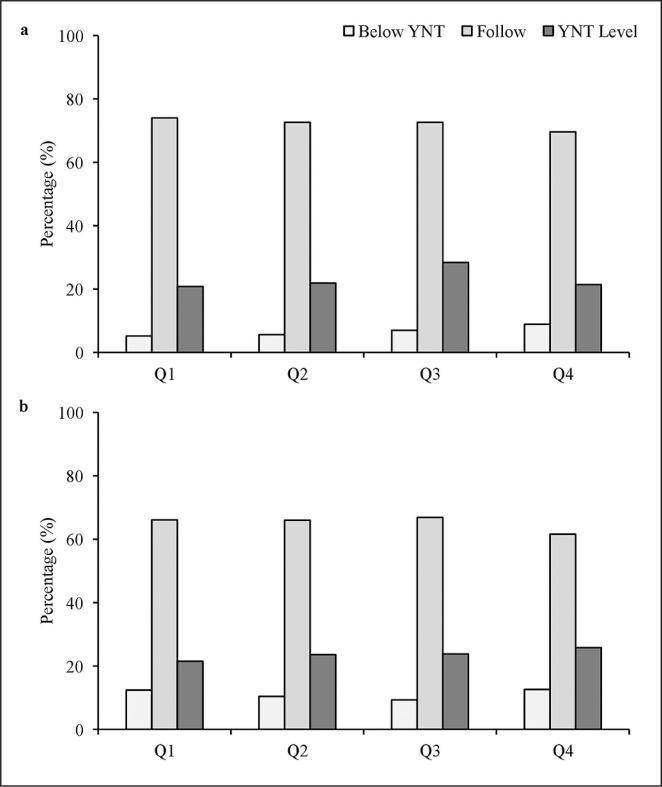
BQ distribution for Club (a) and TID Center (b) presented as a function of potential rating. YNT = Youth National Team

The frequency and percentage distributions of players’ BQs for current performance and potential ability are presented in Table 4. For current performance, in both *Club* and *TID Center*, the chi-squared indicated significant deviations for all current performance ratings, with Q1 players being over-represented and the ORs being relatively similar across groups. Q3 were least likely to attain the highest current rating (‘YNT level’) at *Club* and *TID Center*. A greater % of Q4 players were provided the highest rating, compared to at Club level 21.4% v 15.5%). For potential ability, the chi-squared indicated there were significant deviations for the middle and highestrated players in *Club*, with Q1 players being over-represented. For the lowest-rated players, the largest distribution was observed in Q1, yet this did not reach significance. At *TID Center,* the chi-squared indicated significant deviations for all potential ability ratings, with Q1 players being over-represented. With the recognition that there were unequal numbers of players represented from the BQs, the descriptive percentage results of current performance and potential ability for each BQ at *Club* and *TID Center* are presented. For current performance (Figure 2), Q3 were least likely to attain a ‘YNT level’ rating at *Club* (2a) and *TID Center* (2b). At *TID Center*, more Q4 players were rated as ‘YNT level’. For potential ability (Figure 3), Q4 players were more likely to be rated as ‘below YNT’ but also more likely than Q1 to be rated as ‘YNT level’. At *TID Center*, Q4 were more likely to be rated as ‘YNT level’. Q3 were least likely to be rated as ‘YNT level’ rating at *Club* (3a) and *TID Center* (3b).

**TABLE 4:** Birth quartile distribution by current and potential skill rating.

	n	Birthdate Distribution (%)				Odds Ratio (95% CI)				χ^2^	p	w

		Q1	Q2	Q3	Q4	Q1 vs. Q2	Q1 vs. Q3	Q1 vs. Q4	S1 vs. S2			
**Overall Rating**												

*Club*												
Below YNT	617	224 (36.3)	166 (26.9)	143 (23.2)	84 (13.6)	1.4 (0.7–2.9)	1.7 (0.8–3.7)	2.7 (1.2–6.4)	1.8 (1.0–3.2)	11.80^*^	0.01	0.34 (Medium)
Follow	1265	443 (35.0)	368 (29.1)	290 (22.9)	164 (13.0)	1.2 (0.6–2.6)	1.7 (0.8–3.6)	2.8 (1.2–6.5)	1.9 (1.1–3.3)	11.77^*^	0.01	0.34 (Medium)
YNT level	58	24 (41.4)	17 (29.3)	8 (13.8)	9 (15.5)	1.4 (0.7–3.0)	3.3 (1.4–7.5)	2.7 (1.2–6.2)	2.5 (1.4–4.5)	22.71^*^	0.00	0.48 (Medium)

*TID Center*												
Below YNT	420	154 (36.7)	114 (27.1)	94 (22.4)	58 (13.8)	1.4 (0.7–2.9)	1.8 (0.8–3.0)	2.7 (1.2–6.3)	1.8 (1.0–3.3)	12.28^*^	0.01	0.35 (Medium)
Follow	725	251 (34.6)	221 (30.5)	169 (23.3)	84 (11.6)	1.2 (0.6–2.4)	1.6 (0.8–3.5)	3.1 (1.3–7.3)	2.0 (1.1–3.5)	11.77^*^	0.00	0.37 (Medium)
YNT level	42	15 (35.7)	12 (28.6)	6 (14.3)	9 (21.4)	1.3 (0.6, 2.7)	2.7 (1.2–6.2)	1.7 (0.8–3.7)	1.9 (1.1–3.3)	12.15^*^	0.01	0.35 (Medium)

**Potential Rating**												

*Club*												
Below YNT	121	36 (29.8)	31 (25.6)	31 (25.6)	23 (19)	1.2 (0.6, 2.6)	1.3 (0.6–2.7)	1.6 (0.7–3.6)	1.3 (0.7–2.3)	2.76	0.43	0.17 (Small)
Follow	1410	511 (36.2)	400 (28.4)	320 (22.7)	179 (12.7)	1.3 (0.6, 2.8)	1.7 (0.8–3.7)	2.9 (1.3–6.9)	1.9 (1.1–3.4)	13.13^*^	0.00	0.36 (Medium)
YNT level	409	144 (35.2)	120 (29.3)	90 (22.0)	55 (13.4)	1.2 (0.6, 2.6)	1.7 (0.8–3.8)	2.7 (1.2–6.3)	1.9 (1.1–3.4)	11.92^*^	0.01	0.35 (Medium)

*TID Center*												
Below YNT	132	52 (39.4)	36 (27.3)	25 (18.9)	19 (14.4)	1.5 (0.7, 3.1)	2.3 (1.0–5.0)	2.8 (1.2–6.4)	2.1 (1.2–3.7)	16.43^*^	0.00	0.41 (Medium)
Follow	779	277 (35.6)	229 (29.4)	180 (23.1)	93 (11.9)	1.2 (0.6, 2.6)	1.7 (0.8–3.6)	3.1 (1.3–7.3)	1.9 (1.1–3.4)	13.48^*^	0.00	0.36 (Medium)
YNT level	275	90 (32.7)	82 (29.8)	64 (23.3)	39 (14.2)	1.1 (0.5, 2.4)	1.5 (0.7–3.3)	2.4 (1.0–5.5)	1.7 (1.0–3.1)	9.00^*^	0.03	0.30 (Medium)

TID = talent identification; YNT = Youth National Team; Q1 = Jan-Mar; Q2 = Apr-Jun; Q3 = Jul-Sep; Q4 = Oct-Dec, S1 = Jan-Jun, S2 = Jul-Dec, aQ1 = Aug-Oct; Q2 = Nov-Jan; Q3 = Feb-Apr; Q4 = May-Jul, and semesters: S1 = Aug-Jan; S2 = Feb-Jul, χ^2^ = Chisquared,

* Significant at an alpha level of p < 0.05, w = Cohen’s w effect size.

## DISCUSSION

This study investigated RAE prevalence of youth female soccer players in the US across three stages of the TID process. The main findings were: (1) RAE was present in *Club* and *TID Center*, but not in *YNT*; (2) RAE existed for most positions in *Club* and *TID Centers* (with the consistent exception of full backs across both stages, and wide forwards in the latter). At *YNT*, RAE was only evident in goalkeepers: (3) and RAE was evident in players estimated as early- or on-time-maturers, but not in late-maturing players in *Club* and *TID Centers, YNT* had a greater percentage of late-maturing players, with a reverse RAE. Differences emerged along the TID process, which underlines the value of taking a broader lens when trying to understand RAE in a particular context.

Our data indicated RAEs for *Club* players, with Q1 players overrepresented between U13–U17. Similar patterns were observed at *TID Center*, with Q1 over-represented from U13–U16, Q2 being the dominant quarter at U17, and Q3 and Q4 being over-represented at U18. At *YNT*, RAEs were observed at U15–U16 yet this didn’t follow the typical patterns, with Q2 and Q3 being over-represented. RAE increased from youth-to-senior transitions in female soccer players in Germany [21], yet this was not observed in the US, with U16 *YNT* showing bias to S2 players. Between-country examinations showed no RAEs in U17 players in Europe (11), yet youth players in North and Central America displayed RAEs [13, 24], highlighting the contextual nature of RAE. One of the strongest RAEs was observed in BY2009 at both *Club* and *TID Center* and is in-line with Korgaokar et al. [28] who observed RAEs in US youth female soccer players between 2012–13, yet they only examined one league platform.

More competitive game/platform structures with increased competition for places can lead to more pronounced RAEs at all levels, resulting in the potential for talented players to be overlooked [10]. Therefore, it is possible that an emphasis on earlier-born players is facilitated by the competitive, multi-platform landscape of youth female soccer in the US, as well as opportunities for players to be exposed to college scouts (the next step of the talent pathway) and highly lucrative athletic scholarships may pressure club-level coaches to achieve instant success (playoffs). Although relationships between RAE and success (e.g., final league position) in youth male soccer players in Germany have been reported [33], this was not the same for female national players [13], whilst Andrew et al. [11] reported significant RAEs for U19 players who did not qualify for European Championships. The effect size of RAE decreased from U13–U17, yet it is unclear whether this was due to the levelling of certain advantages, or an overall strengthening of RAEs in female soccer in the last decade [34]. As in our *Club* and previous data [30], scouts may be selecting from an already unequal sample, thus increasing the possibility of RAEs at international levels [24], yet the bias for selecting Q1 players continued to *YNT* for U15 players only.

When analysing RAE and playing position, our data indicated RAEs for midfielders and center forwards with medium effect size, and center backs with a large effect size at *Club* and *TID Center*, with an overrepresentation of Q1 players, yet no RAE at *YNT*. Previous examinations of the role of playing position have reported RAE is most prevalent in female goalkeepers and defenders in Spain [18], defenders and midfielders in Italy [19], only forwards in Olympic teams [12]. Like U17 female players in Italy [22], Q1 midfielders were three times more likely to be selected vs. Q4, yet as previously suggested [13] and consistent with YNT position profiles, we made distinctions between wide and central positions that may have influenced the results. For goalkeepers, RAE was observed, with an overrepresentation Q2 players. Whilst this was only evident in this position, it is consistent with female goalkeepers in Spain [18] and youth players in Europe, North/Central America [24]. It has been suggested that RAE in goalkeepers may be underpinned by a preference for ‘taller’ players [16], but we did not measure stature. A possible explanation could be that it is associated with early physical development being a socially constructed disadvantage for female athletes during puberty and may result in higher disengagement from Q1 players [23: 35] yet the current data showed higher levels of *YNT* labelled as early-maturers from Q2.

Maturity status and RAE play an independent and important role in the TID process of youth female soccer players [36]. Overall, our data showed fewer on-time and late-maturing players, and more early-maturing players, indicating preferences at *Club* and *Talent ID Center* for players with advanced physical maturity. The less latematuring players (10.2%) observed was similar when compared to previous observations of youth soccer players (17.5% [36]), yet we observed a greater overrepresentation of early-maturing players (28.9% vs. 18.3% [36]). These findings may be related to the accuracy of the non-invasive methods utilised within the present study, yet moderate agreement between invasive and non-invasive methods for assessing maturation have been reported from youth male soccer players [37]. Whilst coaches have been shown to be good at judging biological maturation relative to chronological age [29], the high level of early-maturing players at *Club* in the present study may be due to their respective coach’s selection being focused on current over future performance [13]. Regarding biological maturation, early-maturing male soccer players have previously been reported to be ‘taller’ and ‘heavier’ than late-maturing players [38]. Because of the constraints of youth male soccer competition, early-maturing players are able to exploit their physical advantage and progress through the talent pathway [38–40]. In comparison, in youth female soccer players, whilst this seems to be the case at *Club* and *TID Centers*, there were more late-maturing players at *YNT*. This is noteworthy as our sample includes U15–U23 players and does not include the typical ages where maturity differences are greatest in youth female soccer [41] and may be related to recent investments in TID education at *YNT* within US Soccer. Furthermore, the data identified a consistent RAE in early- and on-time-maturing players across the TID process, including an OR of 5.1 between Q1 and Q4 being an early-maturing player at *TID Center*. No RAE was evident for late-maturers within *Club* and *TID Center*, with an RAE reversal [42]) evident in *YNT* players, with late-maturers more likely to be from BQ4 and this group consisted of a higher percentage of late-maturers than from elsewhere in the TID process.

The analysis of current performance and potential ability provides more information on the mechanisms of RAE in youth female soccer. Studies in European female soccer are mixed, with Ginés et al. [36] identifying Q3 and Q4 U12–U14 players as less likely to be perceived as having the potential for future success. Yet Brustio et al. [22] reported that Q4 players were most likely to transition from youth-tosenior international level. Our data indicated that Q3 players had the lowest numbers of current performance rating of ‘YNT level’ (the highest rating possible to achieve), at both *Club* and *TID Center*, yet Q3 players were significantly over-represented at U16 YNT. Ratings for Q4 players were split, with both being most likely to be recommended as the lowest (below YNT) or highest (YNT) levels at *TID Center*. Regarding potential ability, players with the lowest ratings at *Club* were the only non-significant result, they contained less Q1 but more Q4 players than the highest-rated players (i.e., continue to follow/invite to *TID Center*). Relatively younger players may have physical, psychosocial, and motor disadvantages [43] and therefore, to enter and survive in high-performance environments, they may have to acquire higher levels of other skills (e.g., technical/tactical) necessary to overcome RAEs [42]. While relatively older players may not have to possess the same skills to enter the same environment [44], it has been suggested that soccer-nations must give thought to interventions at grassroots (*Club*) level to potentially limit RAEs [45], providing the opportunity for long-term development. It should be noted that US Soccer has recently reformed the TID department (education, courses), thus it would be advantageous to revisit our data to examine the impact of these potential interventions.

## CONCLUSIONS

To conclude, our data showed an RAE of youth female soccer players within the US. At *Club* and *TID Center*, this RAE was most prominent in goalkeepers, center backs, midfielders, and center forwards, and for U13–U18 ages, but these did not typically transfer to *YNT*. Consistent RAEs were observed in early- and on-time-maturers across all levels. A reversal of ‘typical’ RAE was evident at *YNT*, with late-maturers more likely to be from Q4 and a higher percentage than elsewhere in the TID process. When interpreting all the data, some limitations should be acknowledged. Due to the large volume of players and the club soccer environment, we used estimated measurements of maturation. Future research may examine comparisons between scouts’ perceptions and actual biological maturation status. Moreover, we only provided a ‘snapshot’ of RAE in youth female soccer in the US. Therefore longitudinal, cross-sectional analysis examining youth-to-senior transitions would be beneficial to identify if there were different patterns of RAE amongst players retained across the stages in comparison to those newly selected [22].
